# Peripheral blood gene expression profiles linked to monoamine metabolite levels in cerebrospinal fluid

**DOI:** 10.1038/tp.2016.245

**Published:** 2016-12-13

**Authors:** J J Luykx, L M Olde Loohuis, M Neeleman, E Strengman, S C Bakker, E Lentjes, P Borgdorff, E P A van Dongen, P Bruins, R S Kahn, S Horvath, S de Jong, R A Ophoff

**Affiliations:** 1 Department of Psychiatry, Brain Center Rudolf Magnus, University Medical Center Utrecht, Utrecht, The Netherlands; 2 Department of Translational Neuroscience Human Neurogenetics Unit, Brain Center Rudolf Magnus, University Medical Center Utrecht, Utrecht, The Netherlands; 3 Department of Psychiatry, ZNA Hospitals, Antwerp, Belgium; 4 Center for Neurobehavioral Genetics, Semel Institute for Neuroscience and Human Behavior, University of California, Los Angeles, Los Angeles, CA, USA; 5 Department of Pathology, University Medical Center Utrecht, Utrecht, The Netherlands; 6 Department of Clinical Chemistry and Hematology, University Medical Center Utrecht, Utrecht, The Netherlands; 7 Department of Anesthesiology, Intensive Care and Pain Management, Diakonessenhuis Hospital, Utrecht, The Netherlands; 8 Department of Anesthesiology, Intensive Care and Pain Management, University Medical Center Utrecht, Utrecht, The Netherlands; 9 Department of Human Genetics, David Geffen School of Medicine, University of California, Los Angeles, Los Angeles, CA, USA; 10 Department of Biostatistics, Fielding School of Public Health, University of California, Los Angeles, Los Angeles, CA, USA

## Abstract

The blood–brain barrier separates circulating blood from the central nervous system (CNS). The scope of this barrier is not fully understood which limits our ability to relate biological measurements from peripheral to central phenotypes. For example, it is unknown to what extent gene expression levels in peripheral blood are reflective of CNS metabolism. In this study, we examine links between central monoamine metabolite levels and whole-blood gene expression to better understand the connection between peripheral systems and the CNS. To that end, we correlated the prime monoamine metabolites in cerebrospinal fluid (CSF) with whole-genome gene expression microarray data from blood (*N*=240 human subjects). We additionally applied gene-enrichment analysis and weighted gene co-expression network analyses (WGCNA) to identify modules of co-expressed genes in blood that may be involved with monoamine metabolite levels in CSF. Transcript levels of two genes were significantly associated with CSF serotonin metabolite levels after Bonferroni correction for multiple testing*: THAP7* (*P*=2.8 × 10^−8^, *β*=0.08) and *DDX6* (*P*=2.9 × 10^−7^, *β*=0.07). Differentially expressed genes were significantly enriched for genes expressed in the brain tissue (*P*=6.0 × 10^−52^). WGCNA revealed significant correlations between serotonin metabolism and hub genes with known functions in serotonin metabolism, for example, *HTR2A* and *COMT*. We conclude that gene expression levels in whole blood are associated with monoamine metabolite levels in the human CSF. Our results, including the strong enrichment of brain-expressed genes, illustrate that gene expression profiles in peripheral blood can be relevant for quantitative metabolic phenotypes in the CNS.

## Introduction

Gene expression profiling analysis constitutes a powerful tool to increase our understanding of neurobiological mechanisms influencing health and disease, including psychiatric disorders such as schizophrenia,^1^ major depressive disorder,^2, 3^ bipolar disorder^4^ and 22q11 deletion syndrome-associated psychosis.^5^ Integrating transcriptomics with metabolomics approaches may signal neurobiological mechanisms that regulate the metabolome, as demonstrated for the mouse liver.^6^ The selective permeability and cross-talk between central nervous system (CNS) and blood is controlled by the blood–brain barrier. It is currently unknown to what degree gene expression levels in blood may be informative for metabolic processes relevant to the CNS and whether peripheral gene expression levels are linked to CNS metabolism. Although studies comparing transcripts in blood with postmortem brain tissue were recently reviewed,^7^ no investigations relating cerebrospinal fluid (CSF) to peripheral gene expression have been published. Moreover, limited availability of high-quality postmortem brain tissue hampers the applicability of such studies. CSF, on the other hand, allows direct probing of correlations between metabolites in the CNS and peripheral gene expression.

The primary metabolites of the monoamines—5-hydroxyindoleacetic acid (5-HIAA), homovanillic acid (HVA) and 5-medroxy-4-hydroxyphenylglycol (MHPG)—are implicated in physiological CNS processes (ranging from cognitive functioning to reproduction) and neurobehavioral traits, most notably mood disorders^8, 9^ and Brunner syndrome.^10^ Monoamine metabolites (MMs) can be measured in CSF.^11^ Evidence from genome-wide linkage and association studies^11, 12^ attests to the genetic underpinnings of monoamine turnover in CSF.

Here, we examined whether whole-blood gene expression profiles may be informative for the study of CNS metabolomics. Such knowledge may deepen the understanding of the applicability of peripheral gene expression studies for CNS (endo)phenotypes in psychiatry. Moreover, our design has the potential to point to biological pathways implicated in monoamine metabolism, which, in turn, may lead to candidate pathophysiological processes in mental illness. We therefore measured concentrations of MMs in CSF and collected genome-wide gene expression data in 240 healthy human subjects. We not only highlight several genes and gene modules associated with MM levels, but also demonstrate that such genes are enriched for genes expressed in the brain tissue.

## Materials and methods

### Study population

Subject recruitment was described previously.^11^ In brief, volunteers were recruited at outpatient pre-operative screening services in four hospitals in and around Utrecht, The Netherlands, from August 2008 until March 2010. We included patients (i) undergoing spinal anesthesia for minor elective surgical procedures, (ii) ranging between 18 and 60 years of age and (iii) with four grandparents born in The Netherlands or other Northwestern European countries (Belgium, Germany, UK, France and Denmark). Written informed consent was obtained from the participants. Each candidate participant received a personal telephone interview to exclude subjects with self-reported psychotic or major neurological disorders (stroke, brain tumors, neurodegenerative diseases) and to record any use of psychotropic medication.

### CSF collection and monoamine metabolite measurements

From each participant, 6 ml of CSF and 5 ml of blood were collected simultaneously between 0800 h and 1600 h, as described previously.^11, 13^ CSF was kept at 4 °C and transported within 5 h to the laboratory at the University Medical Center Utrecht. The samples were visually inspected for blood contamination and the contaminated samples were excluded from further analyses. Each sample was immediately aliquotted and stored at −80 °C. Concentrations of 5-HIAA, HVA and MHPG were measured using high-performance liquid chromatography with electrochemical detection according to an established method.^13^ Detection failure precluded reliable concentration assessments for 21 MHPG measurements; these were excluded from further analyses. Metabolite concentrations exceeding the mean±3 s.d. were considered outliers and removed (*N*=1 for 5-HIAA and HVA; *N*=2 for MHPG). The MM concentrations were not normally distributed (defined by a Shapiro–Wilk test *P*-value cut-off of 0.05) and therefore log-transformed before analysis. Given the potential increase in power of quantitative genetic analysis of metabolite ratios,^14, 15, 16^ we then also computed the three ratios between metabolite levels.

### RNA quantification procedures, genome-wide expression profiling and quality control

For the isolation and purification of messenger RNA (mRNA) from whole blood, the PAXgene extraction kit (Qiagen, Valencia, CA, USA) was used. The PAXgene tubes were stored at −20 °C and RNA was isolated within 6 months after sample collection according to the manufacturer's protocol, including an optional DNase digestion step. The total mRNA was quantified using a ribogreen assay (Invitrogen Quant-it Ribogreen Thermo Fisher Scientific, Walham, MA, USA). The quality of total RNA was determined using the Agilent 2100 Bioanalyzer (Agilent, Santa Clara, CA, USA). A threshold of RNA integrity number of 7 was applied to ensure good RNA quality. Genome-wide RNA expression profiling was obtained with the Illumina HumanRef-12 (version 3) arrays using Illumina's standard protocol at the UCLA Neuroscience Genomics Core. Raw data extraction and background correction were performed using GenomeStudio (Illumina, San Diego, CA, USA). Quality control was performed by assessment of hierarchical clustering, box plots, density distribution plots and pair-wise correlations (Supplementary Methods). The Lumi package in R was used for robust spline normalization and variance stabilizing transformation of the gene expression data.^17, 18^ A total of 48 803 probes were included on the Illumina microarray. To select those probes that were expressed in at least one sample, a gene filter was applied with the detection *P*-value generated set to 0.01 by GenomeStudio Software (Illumina).^19^ Our gene expression data set consisted of three batches based on Illumina serial numbers. Normalization between batches was performed with the ComBat package for R software.^20^

### Differential expression analysis

The associations of individual genes with mRNA were tested by means of linear regression using the Limma package^21, 22^ ^20, 21^ in R (www.r-project.org). We previously ruled out a significant impact of time of day of sampling, storage duration and the use of psychotropic medication on MM levels,^13^ but age, gender, height and season of the year at the time of fluid collection constitute relevant covariates for CSF MM studies.^13, 23^ For single metabolites and their ratios, a linear regression model was applied including these covariates. The season of the year was encoded as a dichotomous variable (sampling in March, April or May versus the rest of the year).^13^ The gene expression levels were taken as dependent and metabolite levels as independent variables, whereas outcome measures were rate of change (*β*, expression increase or decrease per metabolite level unit). We applied a stringent Bonferroni correction to adjust for the fact that we evaluated 25 361 gene expression probes (that remained after filtering on the basis of quality control criteria) and six phenotypes (all three MMs and their ratios), resulting in a false positive rate of *α*=0.05/(25 361 × 6)=3.29 × 10^−7^.

### Gene network analysis

The 5000 most variably expressed probes^24^ were analyzed using weighted gene co-expression network analysis (WGCNA).^25, 26, 27^ We define co-expression modules as branches of a hierarchical clustering tree; each module was color-coded. To determine a representative module expression profile for each module, we summarized the (standardized) gene expression profiles of the module by their first principal component. This statistic is referred to as the Module Eigengene. The Module Eigengene value of a sample can be thought of as an average standardized gene expression value for all probes in a module per sample. The Module Eigengene is the mathematically optimal summary of a module as it captures the maximum amount of the underlying variation.^28^ We correlated the Module Eigengene of each module with the MMs. WGCNA allows for reconstruction of networks of co-expressed genes and identification of hub genes. Genes with high gene expression significance (that is, highly correlated with the trait of interest) and that are strongly correlated with the Module Eigengene are central hubs and are therefore of interest to monoamine metabolism in our study. Further details are provided in the Supplementary Methods. Again, Bonferroni correction was applied for the 16 modules and six MMs and ratios, resulting in Bonferroni-corrected significance threshold of *α*=0.05/(16 × 6)=5.21 × 10^−4^.

### Functional classification and genetic overlap

A threshold of 10^−3^ was used to select differentially expressed genes resulting from the six association analyses (all three MMs and their ratios). Thus, a total of 1499 Illumina probes were included, corresponding to 1394 unique genes. To determine possible enrichment of this set in genes expressed in brain tissue, we used DAVID (http://david.abcc.ncifcrf.gov/, version 6.7) and the corresponding R package RDAVIDWebService for functional annotation of gene sets included in our analysis.^29, 30, 31^ Databases included to annotate gene list for tissue expression were CGAP EST QUARTILE; CGAP SAGE QUARTILE; GNF U133A QUARTILE; PIR TISSUE SPECIFICITY and UP TISSUE. To address potential biases that can confound this analysis, we also performed a permutation analysis by randomly selecting an equal number of genes and performing DAVID enrichment analysis. Owing to constraints on the use of the DAVID webserver using the package RDAVIDWebService (50 queries per day), we performed 100 permutations. In addition, as RDAVIDWebService does not allow for official gene symbols as input, we used the originally identified Illumina probes as input to the DAVID analysis (*n*=1499).

To assess the possible genetic overlap between the highlighted genes and psychiatric risk genes, we selected genes containing single-nucleotide polymorphisms from genome-wide association studies (with boundaries of each gene being expanded by 20 kb on each side) using the same *P*-value threshold (10^−3^). The threshold of 10^−3^ was picked to be able to select a sufficient number of genes for an overlap analysis, while maintaining a relatively low type I error rate. For this analysis, clumped summary statistics were used from the largest psychiatric genomics consortium (PGC, http://www.med.unc.edu/pgc) genome-wide association studies for major depressive disorder,^32^ bipolar disorder^33^ and schizophrenia^34^ in April 2015. For each trait, these genes were then overlapped with the set of differentially expressed genes, as well as the genes in the modules identified using WGCNA. The significance of overlap with genes in genome-wide association study regions was determined empirically on the basis of 10 000 permutations: for each repetition, a set of genes of the same size was randomly selected from the genes on the expression array and overlapped with psychiatric risk genes. Again, Bonferroni correction was applied for the three psychiatric traits and multiple overlap tests, resulting in Bonferroni-corrected significance threshold of *α*=0.05/(12 × 3)=0.001. As a negative control for this analysis, we also included the latest genome-wide association study results for human height^35^ and performed the same analysis as for the above-mentioned psychiatric phenotypes.

## Results

### Baseline characteristics

Quality control of gene expression data resulted in 233 subjects and 25 361 probes available for analyses. The mean (s.d.) gene expression level of these probes was 8.54 (1.10). Seventy-five percent of the study population was male; the mean (s.d.) age was 39 (11) years. The mean (s.d.) monoamine metabolite concentrations were 147 (62.0) for 5-HIAA, 24.6 (5.07) for MHPG and 211 (74.3) nmol l^−1^ for HVA.

### Differential gene expression analyses

After Bonferroni correction for multiple testing, two genes were significantly associated with monoamine metabolite levels*: THAP7* and *DDX6* at *P*=2.8 × 10^−8^ (*β*=0.08) and 2.9 × 10^−7^ (*β*=0.07), respectively (Table 1); both transcripts were associated with serotonin (5-HT) metabolite levels. For the other metabolite levels, no genome-wide significant results were detected.

### Gene co-expression network analysis

WGCNA allows one to detect co-expression modules, their representatives (known as Module Eigengenes) and the identification of intramodular hub genes. The most significant associations between modules and MM levels were observed for 5-HT metabolism: the Module Eigengenes of the brown module (757 probes) and the turquoise modules (1430 probes) were significantly correlated with levels of 5-HIAA and the ratios 5-HIAA:MHPG and 5-HIAA:HVA (Table 2). The black module exhibited only nominally significant evidence of association with monoamine levels. None of the other modules had a significant association with metabolites or related ratios.

**Figure 1:**
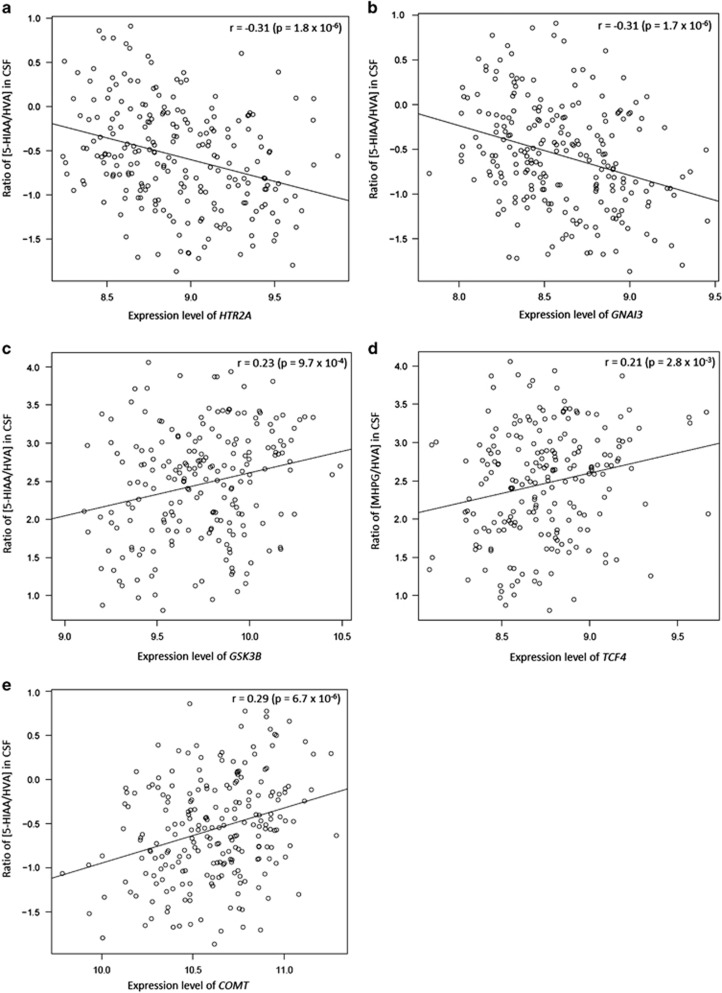
Depicted are the five genes within modules that were significantly associated with monoamine metabolites and that have been implicated in psychiatric illness or monoamine metabolism. (**a**) Correlation between the 5-HIAA/HVA concentration ratio in CSF and expression levels of the gene *HTR2A*. (**b**) Correlation between the 5-HIAA/HVA concentration ratio in CSF and expression levels of the gene *GNAI3*. (**c**) Correlation between the 5-HIAA/HVA concentration ratio in CSF and expression levels of the gene *GSK3B*. (**d**) Correlation between the MHPG/HVA concentration ratio in CSF and expression levels of the gene *TCF4*. (**e**) Correlation between the 5-HIAA/HVA concentration ratio in CSF and expression levels of the gene *COMT*. Each dot represents one healthy subject. Summary statistics are shown in the figures. *COMT*, catechol-*O*-methyltransferase; CSF, cerebrospinal fluid.

Genes whose gene expression levels are highly correlated with the Module Eigengenes can be interpreted as centrally located hubs in the module.^28^ Interestingly, four intramodular hub genes in the MM-related modules were previously implicated in monoamine metabolism or psychiatric disorders. Within the brown module, the gene encoding the serotonin 2A receptor (*HTR2A*) and *GNAI3* were among the top four most highly ranking genes that correlated with the 5-HIAA:HVA ratio (*r*=−0.30, *P*=1.5 × 10^−6^; and *r*=−0.31, *P*=1.4 × 10^−6^, respectively; Figures 1a and b and Supplementary 1A). Within the turquoise module, *GSK3B* and *TCF4* were among the genes most strongly correlated with the 5-HIAA/HVA and 5-HIAA:MHPG ratios (*r*=0.23, *P*=4.9 × 10^−4^; and *r*=0.21, *P*=1.6 × 10^−3^, respectively; Figures 1c and d and Supplementary 1B). The expression levels of these genes explain between 4.4 and 9.6% of the variation in CSF monoamine metabolite concentrations. Within the black module, a module with nominally significant evidence of association with monoamine levels, the gene encoding catechol-*O*-methyltransferase (*COMT*) showed a strong correlation with the 5-HIAA:HVA ratio (*r*=0.29, *P*=5.5 × 10^−6^; Figure 1e and Supplementary 1C).

### Brain tissue enrichment

Using DAVID, we found brain tissue enrichment for the 1499 differentially expressed probes (*P*_Bonferroni corrected_=5.98 × 10^−52^; Supplementary 2). This highly significant result is unlikely to be due to a bias in representation of genes on the array as the permutation results using randomly selected sets of genes show no such enrichment of genes expressed in the brain (Supplementary 2). Moreover, performing the same analysis using the 1394 unique genes corresponding to the identified probes yields nearly identical results (Supplementary 2).

We did not find significant evidence for genetic overlap between differentially expressed genes and psychiatric risk genes or genes in loci associated with height (negative control; Supplementary 3). Nonetheless, we detected a nominally significant overlap (permutation test, *P*=0.04) between genes within the black co-expression module and genes located at schizophrenia susceptibility loci.

## Discussion

Our results demonstrate that gene expression profiles in whole blood are significantly correlated with monoamine metabolite levels in CSF, most notably the serotonin metabolite 5-HIAA. Moreover, the genes highlighted in our study are enriched for genes expressed in human brain tissue.

To our knowledge, this is the first study to link peripheral transcriptome levels to monoamine levels in the CNS. Single gene correlations in our study were particularly strong for serotonin metabolism: *THAP7* and *DDX6* were both found to be associated with the 5-HIAA:MHPG ratio. *THAP7* on 22q11 is a ubiquitously expressed gene encoding a chromatin-associated, histone tail-binding protein, while *DDX6* is a ubiquitously expressed RNA helicase gene. These genes have not been linked to monoamine metabolism previously. As reported before,^16, 36^ we observed stronger associations when using metabolite ratios instead of single MM levels. We postulate that incorporating metabolite ratios may improve the proportion of signal compared with noise by correcting for known and unknown covariates at the individual level.

Our systems biology analysis of the gene expression data using WGCNA managed to identify two co-expression modules that are significantly associated with metabolite levels in CSF. Several intramodular hub genes of these modules have previously been associated with psychiatric (intermediate) phenotypes (Figure 1). For example, *HTR2A* encodes the 5-hydroxytryptamine 2A receptor, a major postsynaptic target for serotonin in the human brain that has been functionally linked to psychosis and antipsychotic response.^37^ In the module that is significantly correlated with the 5-HIAA:MHPG ratio, the hub genes *GSK3B* and *TCF4* seem particularly relevant to neuropsychiatric disease. *GSK3B* encodes glycogen synthase kinase 3β, an enzyme inhibited by lithium.^38, 39^ Preclinical and clinical genetic data implicate the gene in lithium-sensitive behaviors in mice.^38^ *TCF4* encodes a basic Helix-Loop-Helix transcription factor that is highly expressed in human brain and has been implicated in schizophrenia.^34, 40, 41, 42^ Moreover, *TCF4* is in a locus that has been shown to contribute to the shared genetic risk of five major psychiatric disorders.^43^ Although not surviving stringent correction for multiple testing in our study, we find nominal evidence for a link between the prime dopamine metabolite (HVA), the black module and schizophrenia. Within this module, *COMT,* known to encode a paramount dopamine and norepinephrine-degrading enzyme, is one of the most strongly associated hub genes.

Although the mechanisms underlying the association between gene expression in blood and monoamine metabolism in CSF have yet to be uncovered, several pathways that connect peripheral with central nervous system tissues may have a role. Serotonin is mainly synthesized in intestinal enterochromaffin cells and transported into blood platelets by the serotonin transporter (*SLC6A4*).^44^ The transporter in blood is structurally and functionally similar to the CNS transporter.^44^ In blood, these transporters rapidly convey a substantial proportion of peripheral 5-HT into platelets, keeping plasma concentrations low.^44, 45^ In addition, the prime 5-HT catabolic enzyme—monoamine oxidase—degrades both central and peripheral 5-HT. Peripheral and central 5-HT metabolic pathways are thus likely to be highly intertwined or interdependent, which, in turn, may explain why gene expression in blood is informative for CNS monoamine metabolism, in particular, of 5-HIAA.

When interpreting the results, several limitations should be borne in mind. First, a replication set was lacking for the current analyses, which is due to the laborious and invasive nature of collecting CSF. Second, we did not measure the blood:CSF albumin ratio, precluding us from making inferences about the integrity of the blood–brain and blood–CSF barriers. Third, we cannot rule out a (partially) peripheral origin of monoamine metabolites measured in CSF. Examining gene expression in other cell types with a more direct link to CNS (for example, from olfactory cells^46^) may help deciphering this complexity. Future studies that take into account these considerations will provide a more refined understanding of the interplay between CNS metabolism and regulation of peripheral gene expression, which is likely controlled by the blood–brain barriers. Other advancements that will benefit future studies include a larger sample size (to increase statistical power for discovery), the application of high-throughput RNA sequencing (to obtain high-resolution transcriptome data, including in CSF itself), and collection of metabolomic data of both CSF and plasma (to acquire a more elaborate selection of metabolites). The role of the blood–CSF barrier and the interaction between peripheral systems and CNS should also be investigated in patients suffering from mood disorders, Parkinson's disease and other neurodegenerative disorders.^47, 48, 49^

On the basis of our unique data set, we find significant relationships between gene expression levels in whole blood and monoamine levels in CSF. Our finding that gene expression in blood can be linked to monoamine metabolite levels in CSF provides evidence that gene expression profiles in blood constitute a non-CNS component relevant to quantitative metabolic phenotypes of the CNS. Whether this connection across the blood–brain barrier is causally involved in CNS function and pathophysiology (for example, mental illness) remains to be discovered.

URLs used: http://david.abcc.ncifcrf.gov; http://www.med.unc.edu/pgc

## Figures and Tables

**Table 1:** Significant results from the differential expression analysis highlights two genes

*Differential expression associations with the 5-HIAA:MHPG ratio (*N*=202 subjects)*			
*Gene*	*Average gene expression*	β^a^	*Unadjusted* P*-value*
*THAP7*	8.6	0.081	2.8 × 10^−8^
*DDX6*	8.1	0.069	2.9 × 10^−7^

a Log fold changes of expression (expression increase or decrease per metabolite level unit).

**Table 2:** Module Eigengene Pearson correlations with CSF monoamine metabolites levels

*Module Eigengene*^a^ *correlations with monoamine metabolite levels*			
*Monoamine metabolite levels*	*Co-expression module*	r	*Unadjusted* P*-value*
5-HIAA:HVA ratio (*N*=231 subjects)	Brown module (757 transcripts)	−0.28	2.0 × 10^−5^
5-HIAA:MHPG ratio (*N*=209 subjects)	Brown module (757 transcripts)	−0.27	9.9 × 10^−5^
5-HIAA:MHPG ratio (*N*=209 subjects)	Turquoise module (1430 transcripts)	0.25	3.3 × 10^−4^

Abbreviation: CSF, cerebrospinal fluid.

a The Module Eigengene is defined as the first principal component of the module.
