# Supplementary material for: Peripheral blood gene expression profiles linked to monoamine metabolite levels in cerebrospinal fluid
Source: Transl Psychiatry. 2016 Dec 13;6(12):e983–. doi: 10.1038/tp.2016.245 (PMC5290339; doi:10.1038/tp.2016.245)
Supplement: Supplementary Methods [file tp2016245x1.docx]

**Supplemental methods**

Quality Control of Expression Data

*Outlier detection* based on sample distance to center. Outliers are defined as having a sample distance to center > 1.75 SD (depicted by red line) and removed.


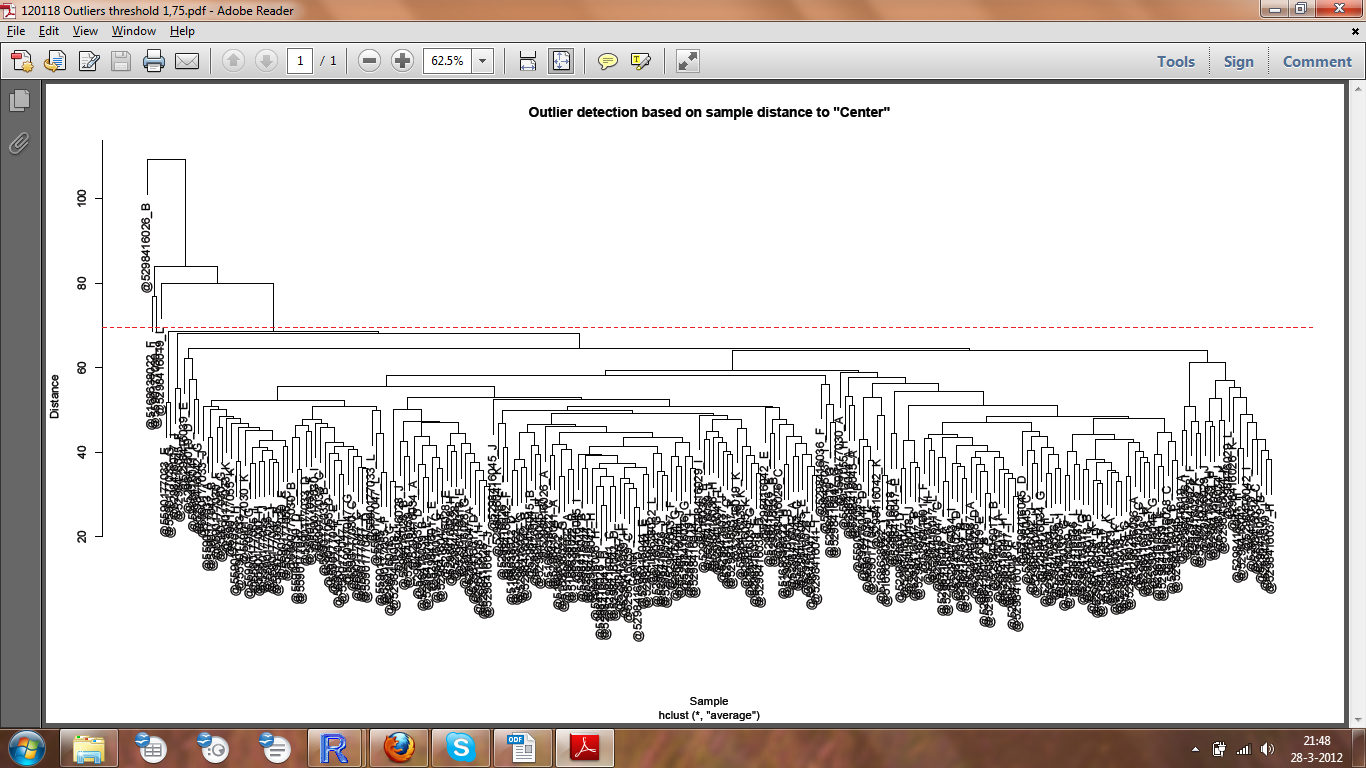


*
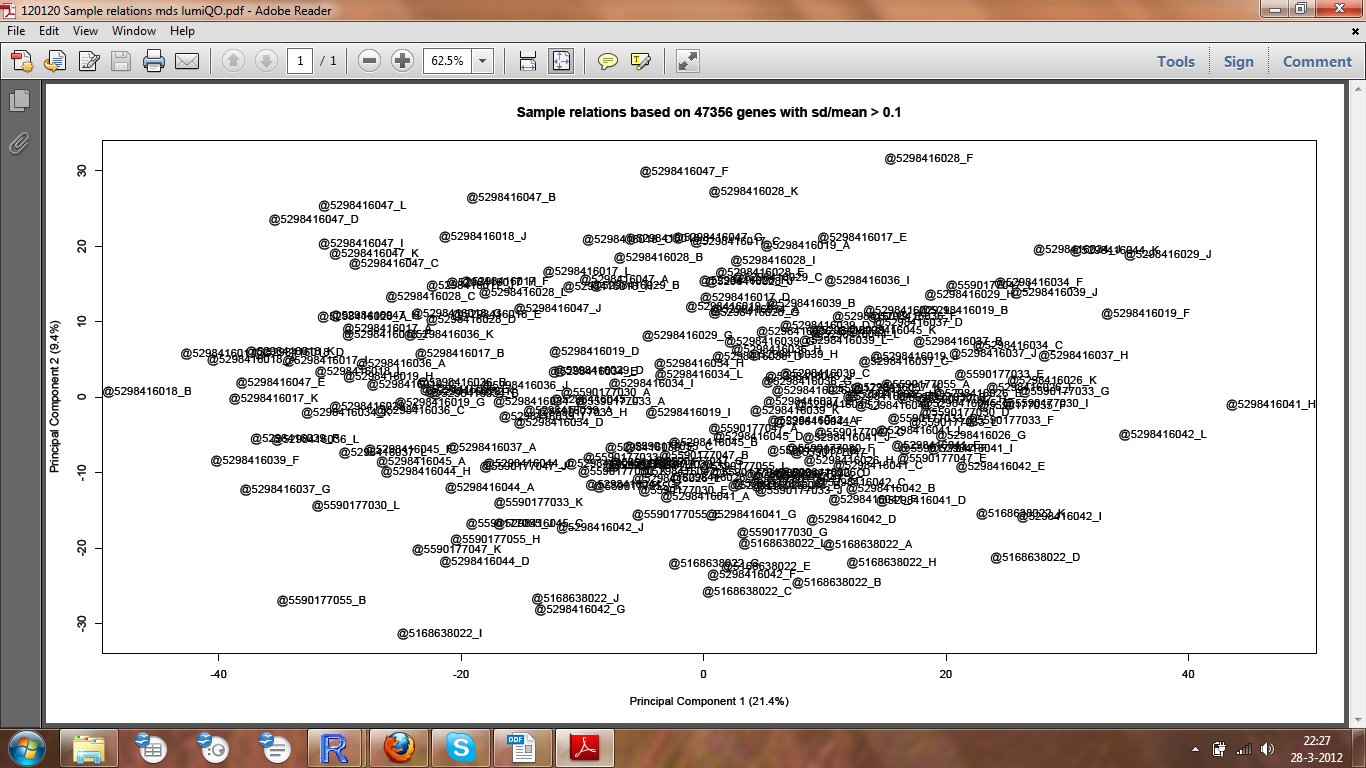
Sample relations*: multidimensional scaling (MDS) plot, showing the first (x-axis) and the second (y-axis) principal component after removal of outliers.

Weighted gene co-expression analysis

A signed weighted gene co-expression network was constructed based on the matrix of pairwise Pearson correlation coefficients. This matrix was raised to a fixed power (β=12 in this study) by the criterion described by Zhang and Horvath ^1^. This threshold resulted in a weighted adjacency matrix containing signed pairwise connection strengths. Specifically, we used average linkage hierarchical clustering with a dissimilarity measure derived from the topological overlap matrix to define a cluster tree. This topological overlap measure is calculated based on the number of shared neighbors. A dendrogram is produced by hierarchical clustering of 1 - topological overlap; branches of the tree are cut using a dynamic tree cut algorithm to define modules (see below). For branch cutting (module detection) we used the dynamic branch-cutting algorithm implemented in the dynamicTreeCut and WGCNA R library ^2^. Each module (or branch) was subsequently assigned a numeric label based on module size, which was visualized underneath the cluster tree. To define a representative module expression profile for each module, we summarized the (standardized) gene expression profiles of the module by their first principal component. This statistic is referred to as the module eigengene. The module eigengene can be thought of as an average gene expression value for all probes in a module per sample and can subsequently be used in association analyses with the phenotypes of interest.

*Network analysis cluster dendrogram* of the 5000 most varying genes, deep split 1. First row: dynamic tree cut in modules. Second row: modules with a between-module correlation >0.8 are merged. For a detailed explanation of the analysis parameters we refer to the work by Zhang and Horvath ^1^.

*
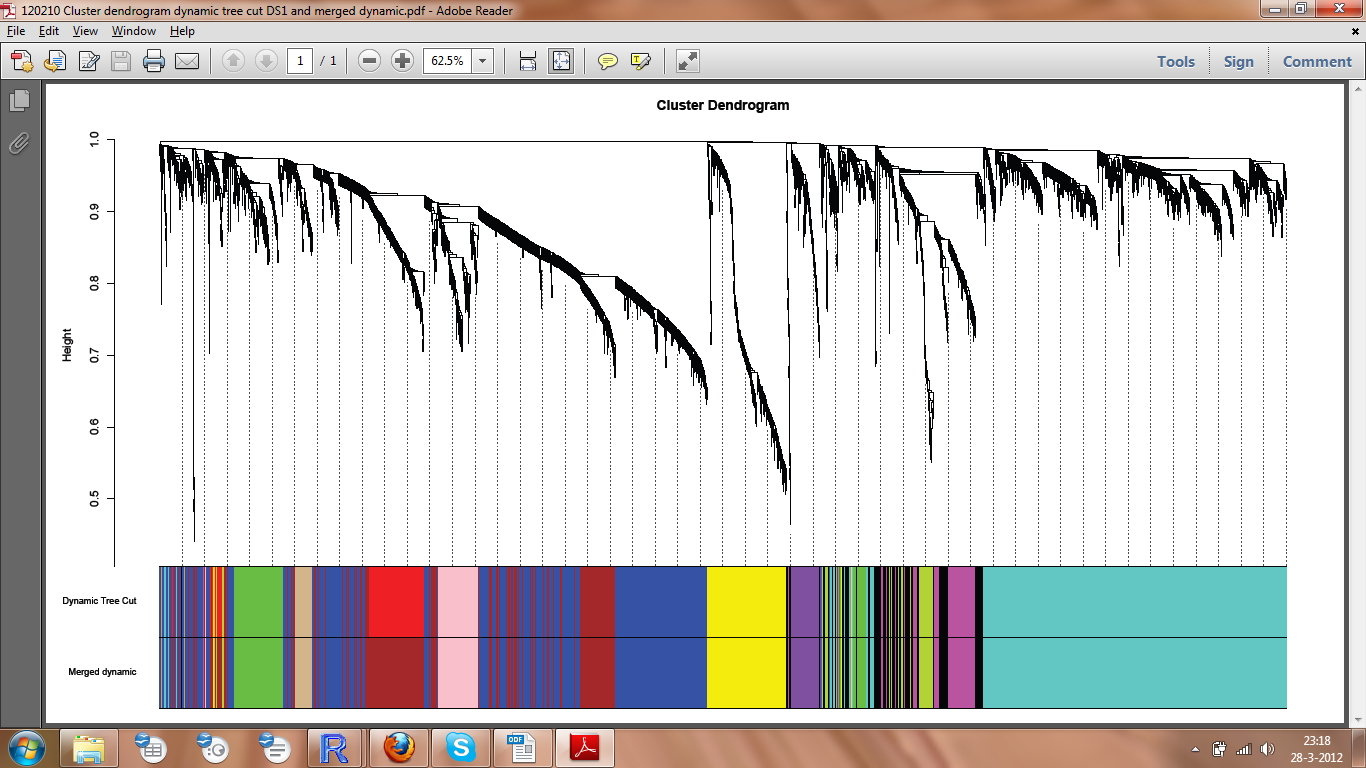
*

1. Zhang B, Horvath S. A general framework for weighted gene co-expression network analysis. *Statistical applications in genetics and molecular biology* 2005; **4:** Article17.

2. Langfelder P, Zhang B, Horvath S. Defining clusters from a hierarchical cluster tree: the Dynamic Tree Cut package for R. *Bioinformatics* 2008; **24**(5)**:** 719-720.
