# Supplementary material for: Peripheral blood gene expression profiles linked to monoamine metabolite levels in cerebrospinal fluid
Source: Transl Psychiatry. 2016 Dec 13;6(12):e983–. doi: 10.1038/tp.2016.245 (PMC5290339; doi:10.1038/tp.2016.245)
Supplement: Supplementary File 1 [file tp2016245x2.docx]

**Supplement 1**Shown are the biologically plausible genes in the modules that stand out in the weighted-gene co-expression analysis (WGCNA). Each selected module contains genes that were previously associated with psychiatric disease, having a high Pearson correlation with both the first principle component of the module (the module eigengene ME) and the monoamine metabolite (MM) ratio. Each dot represents a gene. The brown (A) and turquoise (B) modules show significant associations with monoamine metabolites, whereas the black module (C) does not.


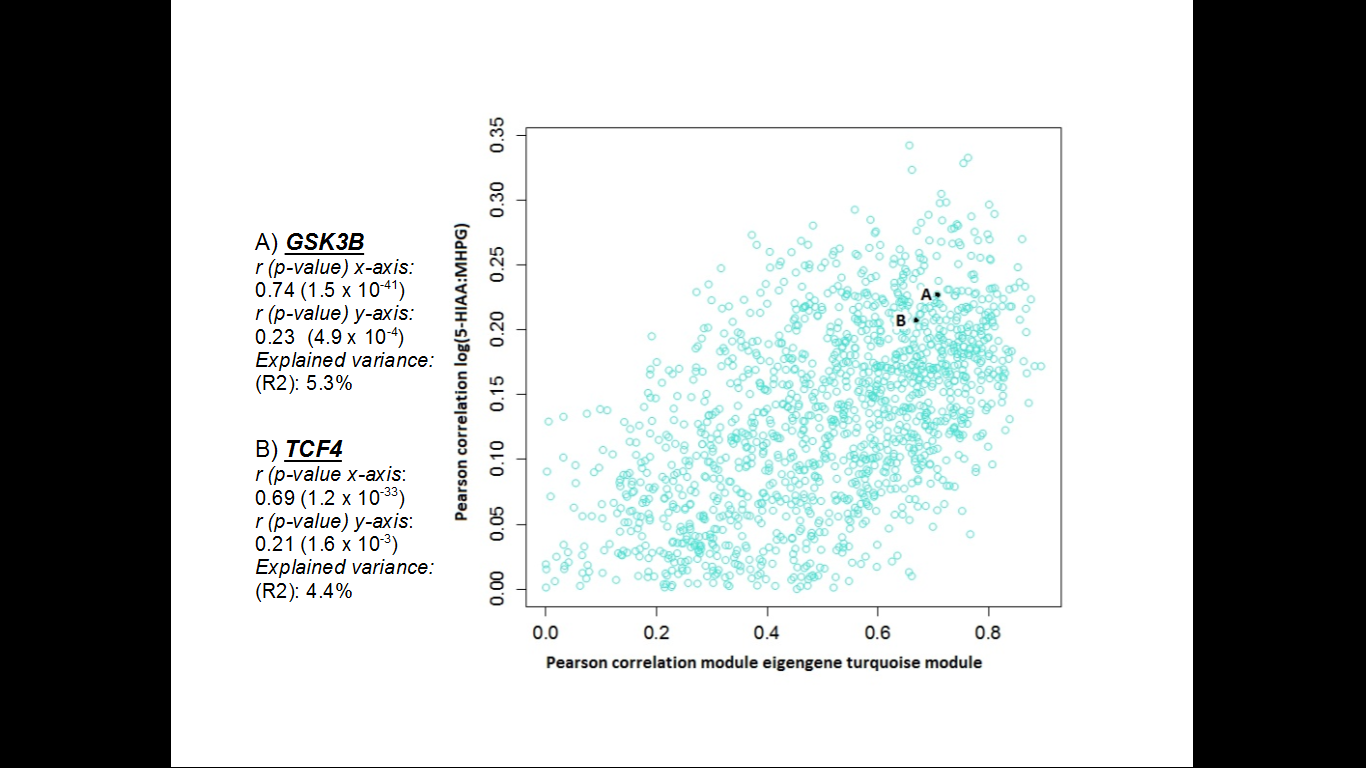

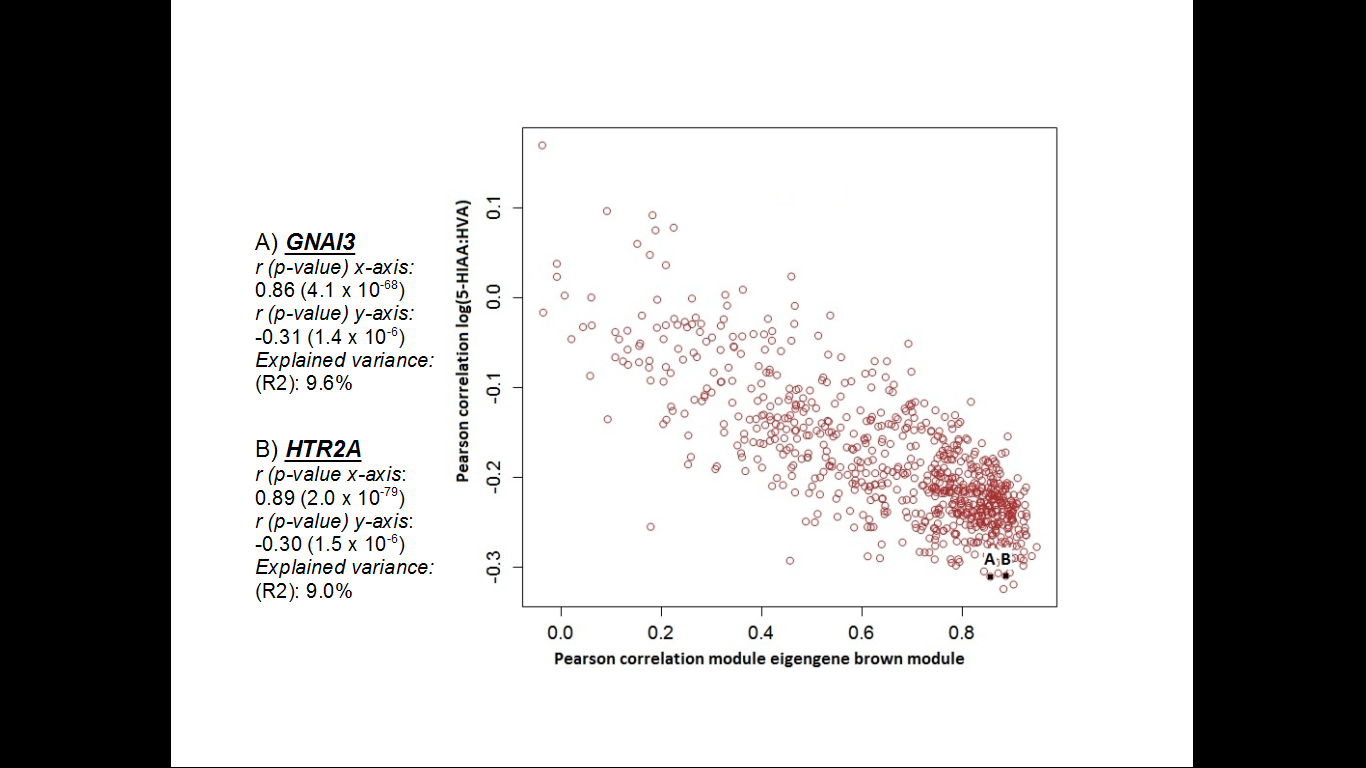
A. ME correlation (x-axis) versus MM correlation (y-axis) of genes in the brown module.

B. ME correlation (x-axis) versus MM correlation (y-axis) of genes in the turquoise module.


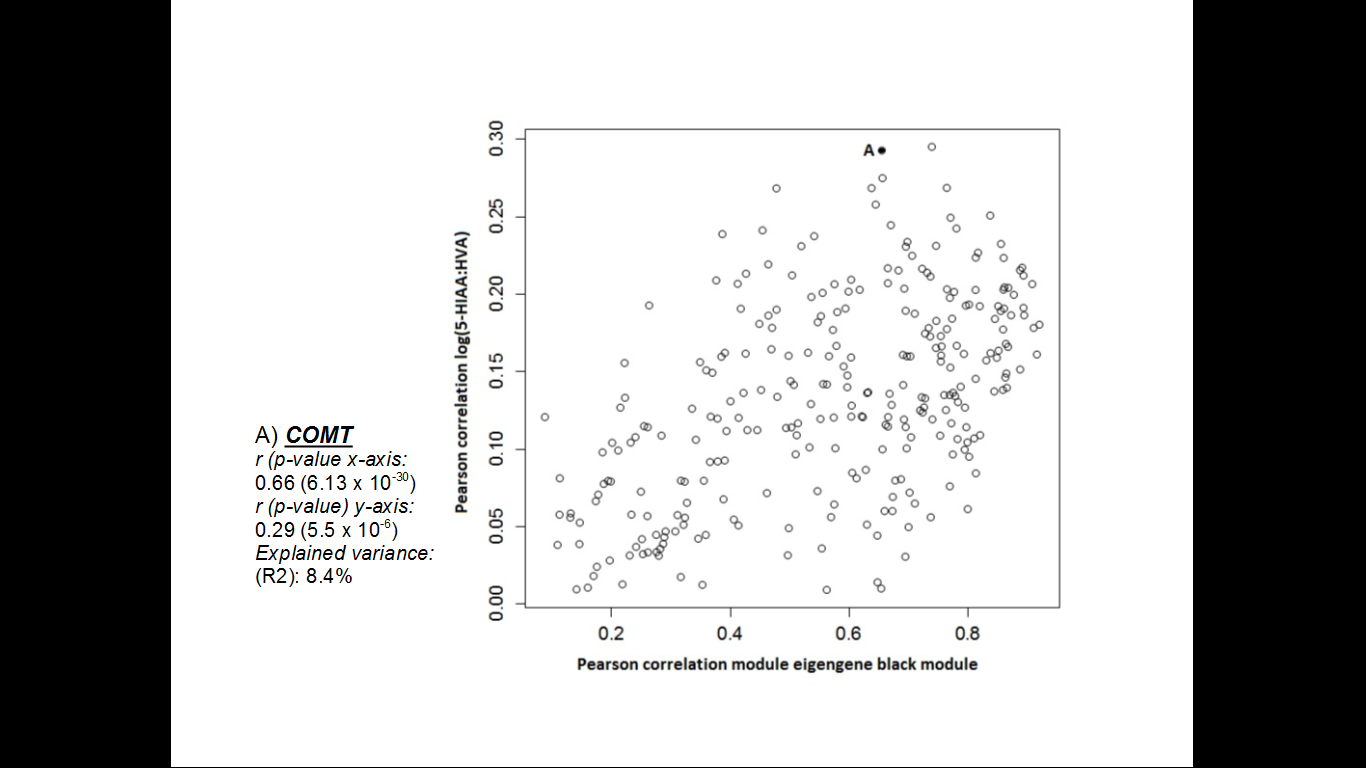
C. ME correlation (x-axis) versus MM correlation (y-axis) of genes in the black module.
